# Patient-Provider Communications in Outpatient Clinic Settings: A Clinic-Based Evaluation of Mobile Device and Multimedia Mediated Communications for Patient Education

**DOI:** 10.2196/mhealth.3732

**Published:** 2015-01-12

**Authors:** Benjamin Schooley, Tonia San Nicolas-Rocca, Richard Burkhard

**Affiliations:** ^1^University of South CarolinaColumbia, SCUnited States; ^2^San Jose State UniversitySan Jose, CAUnited States

**Keywords:** patient-centered information systems, eHealth, mobile computing, medical informatics, patient education

## Abstract

**Background:**

Many studies have provided evidence of the importance of quality provider-patient communications and have suggested improvements to patient understanding by using video-based instruction.

**Objective:**

The objective of this study was to understand how mobile information technology assisted video and three-dimensional (3D) image instruction, provided by a health care worker, influences two categories of outcome: (1) patient understanding of information about their condition and detailed medical discharge instructions; and (2) patient perceptions and attitudes toward their health care providers, which included physicians, nurses, and staff. We hypothesize that video and 3D image instruction, provided on a mobile, tablet hardware platform, will improve patient understanding about the diagnostic testing, diagnoses, procedures, medications, and health topics provided to them. We also propose that use of the tablet/video combination will result in improved attitudinal evaluation by patients of their providers and the treatment plan.

**Methods:**

This study evaluated a hospital clinic-based trial (patient N=284) of video and 3D image instruction, provided on a mobile, tablet hardware platform, and its potential to improve patient understanding about the diagnostic testing, diagnoses, procedures, medications, and health topics provided to them.

**Results:**

Results showed strong evidence that the system was perceived as helpful for improving patient understanding, and that it improved communication between physicians and patients (*P*<.001). The advanced age of some patients had no effect on their perceptions of the tablet-based mediation. Physician comments provided useful insights on effective use of such systems in the future. Implications for further development and future research are discussed.

**Conclusions:**

This study added to the body of evidence that computer-assisted video instructional systems for patients can improve patient understanding of medical instructions from their health care providers and assist with patient compliance. In addition, such systems can be appealing to both patient and provider.

## Introduction

### Research Goals and Objectives

The goal of this study was to understand how video and three-dimensional (3D) image instruction, implemented on a mobile, tablet hardware platform and provided by a health care provider, influences two categories of outcome. The first outcome is patient understanding of information about their condition and the detailed medical instructions to be followed after the patient exits the clinic. The second outcome is patient perceptions and attitudes toward their health care providers, which included physicians, nurses, and staff. We hypothesize that video and 3D image instruction, provided on a mobile, tablet hardware platform, will inform and assist patient understanding about the diagnostic testing, diagnoses, procedures, medications and health topics provided to them. We also propose that use of the tablet/video combination will result in improved attitudinal evaluation by patients of their providers and the treatment plan.

Several studies have provided evidence of the importance of quality provider-patient communications and have suggested the potential for improvements to patient understanding by utilization of video- based instruction. This study extends past research by: (1) focusing on an outpatient clinic patient population across a broad range of conditions (ie, all adult patients visiting an outpatient health clinic), (2) utilizing larger wireless tablet computers to provide for handheld video instruction, (3) assessing use of videos in relation to patient understanding and overall provider satisfaction, and (4) including a larger number of participants than prior studies focusing on mobile video based instruction.

**Figure 1:**
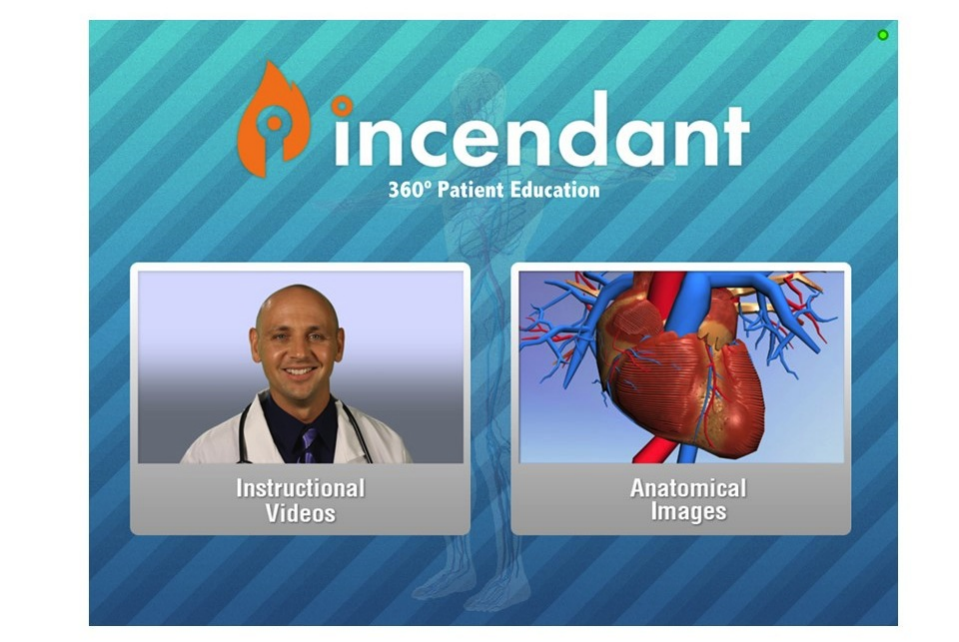
System screenshot 1.

**Figure 2:**
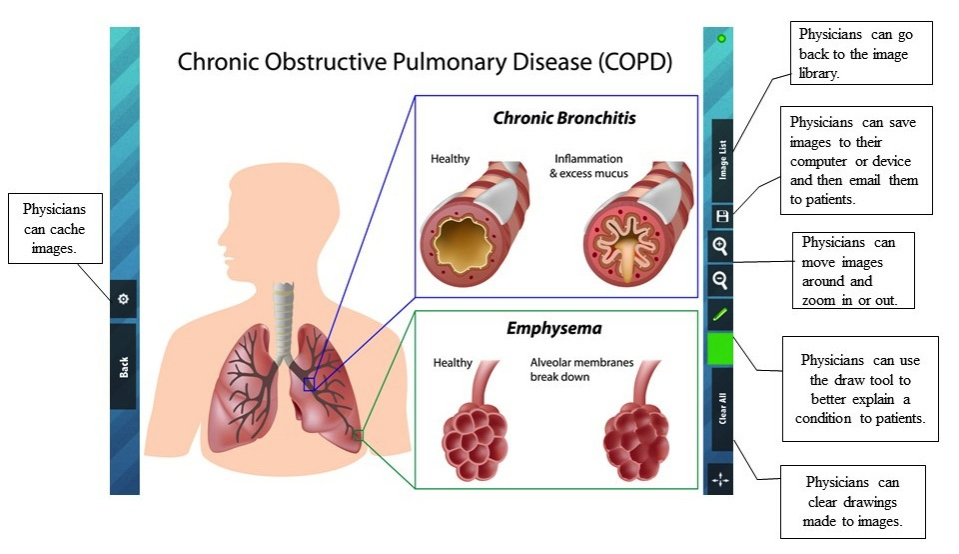
System screenshot 2.

**Figure 3:**
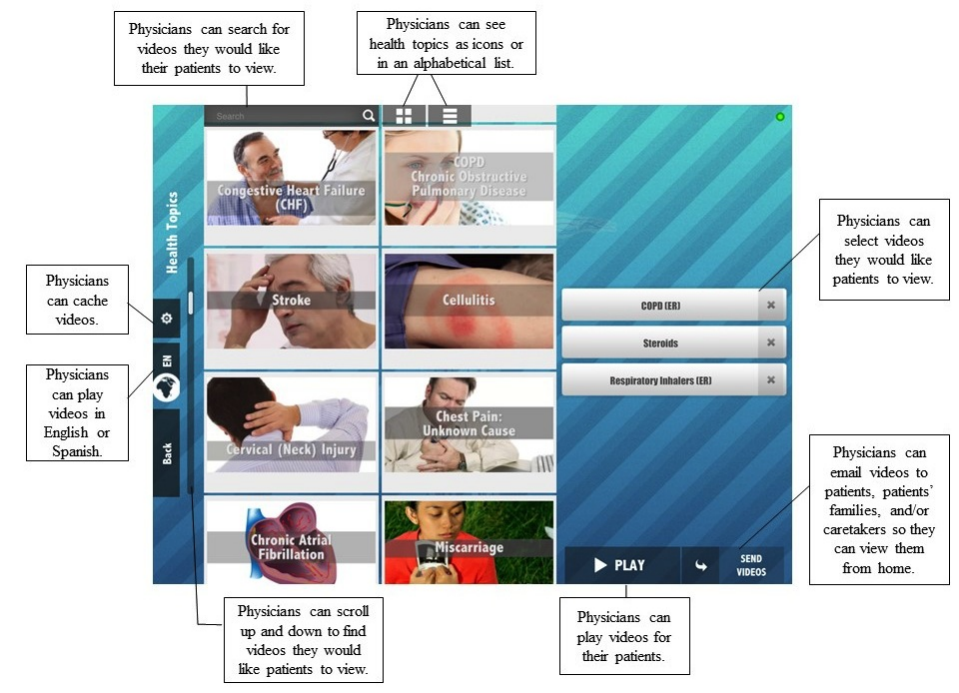
System screenshot 3.

The specific aims of this research were to investigate if video and 3D image instruction, implemented on a mobile, tablet hardware platform and provided by a health care provider, helps health care workers to: (1) assist patient understanding, and (2) help provide a positive overall experience of the provider for patients. The research is intended to test the utility, practicality, and patient-perceived usefulness of video and 3D image instruction presented on a handheld mobile tablet device (Figures 1-3 show illustrations).

### Provider-Patient Communications in Outpatient Settings

Many research efforts since the early 1990s have focused on the potential of information technologies to improve communications between medical providers and patients. Areas in which information technologies have been investigated naturally involve some of the main purposes of patient provider communications. These include creating good interpersonal relationships, facilitating exchange of information, and assisting in the making of treatment related decisions [1]. The importance of these objectives is seen in the fact that the quality of physician-patient relationships have been shown to affect patient recovery from illness [2], the cost of care [3], malpractice claims [4], and patient outcomes for chronic diseases [5,6].

Technology implementations should reflect the recent focus in the provider-patient communication literature on “patient-centered communication”, the idea that health care providers deliver care that is focused on patient needs and preferences, coupled with collaborative medical decision making [7]. Patient-centered behaviors that affect outcomes improve communications by responding to patient concerns and allowing for participatory decision making [8]. An open, communicative relationship between provider and patient in the management of chronic conditions helps patients understand their health conditions and helps reduce patient stress levels [9]. Patient-centered communication is found to have a positive relationship with patient health outcomes, satisfaction, and adherence to instructions in primary care [10]. Physicians who used more patient-centered behaviors inspire greater confidence, as well as greater willingness by patients to accept physician recommendations [9,11].

Technologies that assist communication become part of the environmental setting of provider-patient communications and will affect provider communication behaviors, techniques, and effectiveness. The importance of quality provider-patient communications during discussion about a patient management plans is seen in its influence on several outcomes, including patient emotional health, symptom resolution, body functions, physiologic measures (ie, blood pressure and blood sugar level), and pain control [6]. Positive health outcomes are associated with a wide range of both verbal and nonverbal communication behaviors [12].

A recent report on chronic disease detailed findings that patient education should be directed at improving quality of life, and included tailoring communications to the special needs and environment of the patient. Chronic disease settings, in particular, call for interactive, simple to follow, and practical communications that are appropriate to the intellectual and social skills of the patient and the caregiver [13].

The potential benefits of improved provider-patient communications are profound, patient knowledge and self-efficacy can improve along with increased adherence to the provider instructions and improved patient self-management [7]. When patients perceive providers to communicate clearly, carefully, and thoroughly, patients are more likely to actively participate in their care and make better-informed decisions. Well-informed patients achieve a common understanding with their physicians, and adhere more fully to treatment instructions [5,8].

### Communications at Time of Patient Exit From the Clinic

Technologies intended to assist medical communication are added to an environment in which communication skills and emotional awareness of health care providers are defined as key aspects of professional competence [8]. Unfortunately, increasing pressures placed on medical providers to process patients in a minimal amount of time work against efforts to encourage improved communication between the provider and the patient [4,7]. In time-pressured environments, some providers may regard sensitivity and clarification in communication as a luxury. Nevertheless, the quality and effectiveness of instructions at time of patient exit from the medical facility present critically important opportunities for ensuring continuity of recovery.

As a result, the routine setting of a patient leaving a health care facility at the conclusion of receiving health related services presents responsibilities for health providers to communicate effectively with their patients, that cannot be appropriately substituted with other visits [12]. Patient instructions, for example, in the form of a management plan, care plan, consultation, or ongoing medical maintenance instructions, typically include advice regarding the ongoing management of a clinical condition, medications, complications, and required follow-up [14]. For example, at patient exit from an emergency department visit, the provider must effectively complete three primary tasks: (1) communicate crucial information, (2) verify comprehension, and (3) tailor teaching to areas of confusion or misunderstanding to ensure patient safety in the home environment [15].

The term “discharge” is often applied to inpatient settings. Studies that address inpatient discharge, and the medical instructions at the time of inpatient discharge, provide many insights into the outpatient post treatment setting, especially when patients exit a clinic after treatment. In both cases, patients exit with detailed, often complex, medical maintenance instructions. Much research addressing inpatient discharge is relevant to the outpatient setting of this study.

Successful communication of discharge information is critical, as patient noncompliance with instructions can lead to safety risks for patients after discharge. The range of possible risks includes inappropriate home care, including incorrect medication use, and failure to return for concerning symptoms or follow-up as directed [15]. Such outcomes not only affect the health of the individual, but also the health care system, as patients with poor comprehension are at increased risk for adverse events and increased health care utilization [16].

A number of factors lead to patient noncompliance with discharge instructions. Patients complain that verbal instructions from physicians and/or medical staff are not provided in simple language [17], or a patients’ spoken language [18], and are therefore difficult to understand. In addition, the mean reading level of patients in some studies is reported to be equal to or below a seventh grade level [19,20], while printed discharge instructions are often written at the eleventh grade level [20], or at a college level [21]. This is a significant problem, since the discharge process happens quickly, with many instruction situations averaging a duration of approximately seventy-six seconds [22]. Patients may feel rushed and not think of or ask relevant questions that might help ensure their understanding of discharge instructions.

### Medical Instructions in the Context of Health Literacy

Limited health literacy contributes major challenges to patient comprehension of medical care and self-care instructions. Health literacy includes the cognitive and functional skills needed by a person to make health-related decisions [23,24]. It is estimated that at least 26% of the population has limited health literacy [24,25]. The high prevalence of poor health literacy complicates the discharge process because many patients are not able to fully comprehend written resources [15].

Health literacy issues have added to the perceived importance among those in the medical communities of improving physician-patient communications, with a greater focus on related competencies in recent years. For example, the American Board of Medical Specialties now requires assessment of provider- patient communication for continued certification [7].

Training in communication skills for new physicians is an important part of satisfying recent national expectations for improved primary care, and learning to more effectively share information with patients is a central characteristic of such training [7]. However, newer learning techniques for the patient may also yield great benefits for improved primary care, and the foundation of the current study is that computer-based interaction that is flexible and adaptable to individual patient needs, with appropriate video and other visual enhancements, is one of the most promising methods for improving and reinforcing communication.

### Medical Instructions in the Context of Special Patient Characteristics

Each patient has individual communications needs that may be influenced by several key characteristics: (1) age, including the youngest through the oldest; (2) hearing and speaking capacities; (3) cognitive capacities; and (4) language requirements. In addition, medications may affect ability to understand and retain medical discharge information.

Some studies have shown that patients with cognitive limitations often fail to remember clearly the instructions that health care providers have given them [1], which has obvious outcomes for reduced compliance and treatment effectiveness. Education of patients for recovery of medical intervention should be focused on simplicity, practicality, and messages that are “appropriate to the intellectual and social skills of the patient” [13]. Adherence outcomes are supported by recent experimental evidence that indicates that health care providers who are focused on patient centered communication are seen as more competent and trustworthy than those who are less focused on such communication [11]. Improved patient adherence is an additional benefit thought to be associated with patient centric communications [26]. Patients may see the patient centered communication approach as a “therapeutic alliance” between provider and patient [26], and in these contexts patients may be more comfortable with asking questions and more successful at obtaining information, which can reduce patient anxiety [6].

### Computer-Based Mediation for Older Patients

The ability to manage provider-patient relationships by applying good communication skills is an essential component of health care professional competence [7], and this may be particularly relevant for older patients. Providers may face the need to adapt their communication patterns for higher-risk patients, such as older patients with chronic, often painful conditions, who tend to focus more on biomedical rather than coping concerns in their communications [26].

Older patients encounter a medical environment in which desktop computers are increasingly used in diagnosis and in real time data on the appropriateness of specific prescription medications. As populations in many countries age, use of computers in the doctor-patient encounter can only be expected to increase [27]. Using computers in the patient-physician interaction, rather than office or bedside consultations, can help older patients understand the risks of their condition and how adhering to instructions can reduce their risks.

Older patients may be more accustomed to the verbal reassurances and emotional support that characterize face-to-face patient-provider communications, and in this regard, computer-mediated communications may be less effective than traditional methods [12]. For example, patients are particularly satisfied by empathy and psychosocial talk, but it may be that relatively few health care providers provide these benefits [12]. On the other hand, computers can assist in providing specific directions that are a source of satisfaction to older patients, and can lead to improved compliance in this patient group.

### Visible Interfaces and Videos as Mediators Between Provider and Patient

When patients can see their current patient electronic health records (EHRs) on computer screens, their perceptions of patient provider communication are improved [28]. Computer mediated communications, including the use of the EHR at the patient side, may be a tool to enhance patient-centered communications [28], and recent studies have described ways to integrate the use of an EHR into patient communications [29]. Others have noted how multimedia may be integrated into the EHR and patient care in the future [30].

Use of video instruction for patients about disease treatment and recovery has been studied from at least as early as the mid-1990s [10]. However, while it is common for physicians to use computers for their own work during patient consultations, the delivery of computer-based video instruction directly to patients remains relatively uncommon [31], and the use of handheld devices for such instruction appears to be rare. When computers are introduced into doctor-patient communications, the relationship changes, altering the distribution of power and authority between doctor and patient [32].

Both positive and negative responses to computer mediation have been seen with both providers and patients. For example, general practitioner use of computers during patient consultations sometimes demonstrates a negative effect on patient-provider communications [27]. Other research has found no significant difference between face-to-face only versus face-to-face plus video instruction of post operative ostomy patients in regard to self-care skills or confidence in performing ostomy self-care [33].

Still other studies have suggested that patients view computer-based video use by physicians favorably increasingly often [31,34]. Thus, providers should be aware of the potential for both positive and negative results, and should be aware of usability and effectiveness of computer-based systems when communicating with patients.

### Mobile Devices in Clinical and Patient Settings

As handheld devices for patient monitoring and instruction become increasingly widespread in the United States and Europe [35], smartphones are convenient and available portable devices for patient use, and many studies document experiments with smartphone applications for patients [35].

While smartphones and other mobile devices have been used in medicine to accomplish various tasks, very few high-quality studies have demonstrated appropriate application of this technology, including the few applications specifically designed for patient education [35]. In one study, nurse midwives in India were provided mobile phones to support patient education with the intent of assessing the impact of using mobile devices to provide video during patient encounters. Results showed that changing the process of patient education by using mobile video improved patient education provided by the nurses [36]. Only a small number of publicly available videos through the Internet (n=56) were found to be educationally useful for teaching medical practitioners about physical examination of the cardiovascular and respiratory systems [37].

### Computer-Mediated Video Instruction Versus Traditional Media

Use of instructional videos in medical contexts, as compared with traditional printed document-based instruction, is gradually becoming more common. As educational videos for medical providers on various ailments and treatments have become common on public media channels such as YouTube, video guidance for patients has started to become more available [37]. However, use of free online channels for patient education is fraught with problems, in that a large proportion of such videos are of poor educational value, and the quality of such instruction cannot be assured [37].

It is not yet known how the introduction of instructional videos by health care providers will affect perceptions by patients of the patient-centeredness of providers or satisfaction with provider communications. [11]. There are three studies that have been published in the medical literature about the use of videos to improve patient understanding of provider instructions. Meade et al (1994) investigated whether printed or videotaped information is more effective in enhancing colon cancer knowledge [38], and results suggested that both printed and videotaped materials enhanced colon cancer knowledge among patients with limited literacy skills. Leiner et al (2004) compared the effectiveness of a printed message about polio vaccinations with the same message converted into a production of animated cartoons using marketing and advertising techniques in a pediatric clinic. Results suggested that animated cartoons could improve knowledge among parents or caretakers about the polio vaccination. Choe et al (2009) evaluated the effectiveness of mobile discharge instruction videos in communicating discharge instructions to patients with lacerations or sprains in a prospective, controlled study on patients at an emergency center for two months. Patients received either printed discharge instructions or mobile discharge videos, with mobile discharge videos seen by patients as improving the communication of discharge instructions [34]. Finally, in another study, glyph pictographs were used to illustrate discharge instructions to patients. Discharge instruction recall improved significantly among participants in the test group over those that received nonillustrated instructions [39].

In summary, the body of evidence and field evaluations of the use of computer-based methods to mediate between health care providers and patients raises many important questions and new, unresolved challenges. In addition, the use of computer-based videos to educate patients and the application of handheld, mobile devices in these contexts remains in the early stages of development and testing.

## Methods

### The Study Participants

This study investigated health care worker use of information technology assisted video and 3D image instruction to assess the impact on patient perceptions of helpfulness of the intervention and patient perceptions about their provider. There were two hundred eighty-four patients that were enrolled in the study, of which half (142) were given the information technology (IT)-based instruction, and half (142) were instructed without the IT-based system. The study group consisted of patients receiving video and 3D image instruction via a wireless handheld device provided by a health care worker. The control group consisted of patients receiving ordinary, noncomputer-mediated instruction provided by their health care worker.

### Study Setting

The research was conducted at an outpatient clinic in southern Colorado and all health care workers providing instruction were medical doctors serving as residents at the clinic. The clinic is part of a hospital system with Level II Trauma Care certification, 370 staff physicians, 350 critical care beds, and 2600 employees, serving a local population of nearly 400,000.

### Provider use of Mobile Devices

There were ten first year residents working at the clinic that were provided with standardized training on appropriate physician-patient interactions in regards to providing patient instruction and education. All ten of the residents were provided with an Android tablet wireless mobile device capable of viewing 3D images and video. There were five of the residents that were randomly selected to provide video-based instruction that included 3D images to their patients (the study group) using the handheld wireless devices. The five selected residents were also provided access to a server containing video and 3D instruction material through the clinic’s wireless network. The instructional systems, including, tablets preloaded with videos and 3D images, were provided by Incendant Corporation, a commercial provider of instructional videos and 3D images for use in hospitals, clinics, and home patient education. The videos cover topics of importance to patients, such as diagnostic testing, diagnoses, medical procedures, medications, and health topics. A complete listing of the available topics can be found in Appendix 3 (see Multimedia Appendix 3). The other five residents provided instructions to their patients using traditional, noncomputer mediated methods, such as written or verbal instructions. Residents from both groups chose the specific instruction content for each patient based on his/her determination of the instructional needs for each patient. Figures 1-3 show typical instructional images and videos on the mobile tablets.

### Data Collection and Patient Groups

Any patient who visited and was treated at the clinic during the study period of 180 days was invited to participate in the study. If the patient was under 18 years old, the parent(s) or legal guardian was asked to participate in the study. The total study sample (n=284) was divided into two groups (n=142, each), with one group receiving the discharge videos, and one receiving standard discharge instruction. A set of questions preapproved by the medical center’s Institutional Review Board was used to assess patient views and reactions to the discharge videos, as well as the health care providers, and the medical center. After receiving instruction and educational material from a resident, participants (patients) were asked to complete preselected questions based on the Consumer Assessment of Health Care Providers (CAHPS) Clinician and Group survey instrument for adults and children. The survey also collected data on patient self-perceived general health status, mental health status, age, gender, and education level. Medical assistants administered the paper survey to each patient in a private environment in the facility. Completed surveys were dropped into a locked, secure box at the medical assistant office desk.

Medical assistants administered an additional survey to the medical residents involved in the study (n=7) during a 60 day period. The medical residents completed a paper evaluation survey at their convenience. Evaluation surveys were dropped into the locked, secure box at the medical office desk.

The patient survey and medical resident survey are included as Appendices in this article (see Multimedia Appendices 1 and 2), respectively.

### Hypotheses

Given the support found in the background literature that use of computers, with high visibility interfaces and appropriately designed software, to communicate detailed information in both patient-provider settings and the patient use alone setting, we propose H1.

H1, patients will find the provider’s use of tablet device to communicate information to be helpful.

Prior research has not established whether use of computer devices, with appropriate interfaces and software, facilitates direct verbal communication between patient and provider. However, the background literature supporting H1 suggests the possibility of reasonable extension of expected improved communication to direct conversation between the patient and provider. As such, we propose H2.

H2, patients will find the provider’s use of a tablet device to communicate information to them will make it easier for the patients to talk with providers.

In view of the background literature suggesting an age-related effect on individual ability and preference to use computers, we propose H3.

H3, older patients will find the provider’s use of tablet device to communicate to be less helpful than will younger patients.

## Results

### Primary Results From the Post Medical Appointment Survey

Primary results from the post medical appointment survey of patients are shown in Table 1, below. The total number of participants was 284, split equally between the treatment group in which the provider used the tablet/software bundle in face-to-face communication with the patient group, and the control group, in which the tablet/software bundle was not used. Comparisons between groups are seen in items 2, 3, 4, 5, 6, 7, 8, 9, 15, and 16, as shown in the “Explanation” column in Table 1, below. Comparisons within groups were applied to items 11 and 12. H1 and H2 were evaluated using tests of comparative proportions, with significance provided by Fisher’s exact test. Hypothesis 3 defined older age as 65 and above (37.3% of total sample, 106/284) and younger age as 18 through age 64 (62.3% of total sample, 177/284), and was evaluated using a simple *t* test for comparison of groups.

**Table 1:** Primary survey results, communication effectiveness, both groups.

Item	Assessment question	Response^a^	N=284, n	Percent	Explanation
2	Seen within 15 minutes?	Yes	257	90.8	Question applied to both treatment and nontreatment groups (N=284)
		No	26	9.2	
3	Provider easy to understand?	Yes	279	98.2	Question to both groups
		No	5	1.8	
4	Provider listened carefully?	Yes	282	99.6	Question to both groups
		No	1	0.4	
5	Discuss problems with provider?	Yes	275	97.5	Question to both groups
		No	7	2.5	
6	Provider gave clear instructions?	Yes, definitely	264	97.4	Question to both groups
		Yes, somewhat	6	2.2	
		No	1	0.4	
7	Provider knew important information about patient?	Yes, definitely	247	88.2	Question to both groups
		Yes, somewhat	32	11.4	
		No	1	0.4	
8	Provider respected patient comments?	Yes, definitely or somewhat	279	99.6	Question to both groups
		No	1	0.4	
9	Provider spent enough time with patient?	Yes, definitely or somewhat	278	98.2	Question to both groups
		No	5	1.8	
10	Provider used computer to show information?	Yes	136	48.9	Distinguishes treatment and control groups
		No	142	51.1	
11	Was provider’s use of computer helpful to you?	Yes, definitely	123	85.4	Treatment group only
		Yes, somewhat	16	11.1	
		No	5	3.5	
12	Provider’s use of computer made it easier or harder for patient to talk with provider?	Harder	5	3.5	Treatment group only
		Not harder or easier	50	35.2	
		Easier	87	61.3	

^a^ Total responses to items 2, 4, 5, 6, 7, 8, 9, and 10 do not equal 284 because of nonresponse by subjects.

**Table 2:** Survey results, medical conditions.

Item	Assessment question	Response	N=284, n, (%)	Explanation
15	Overall health	Excellent	22 (7.9)	Question to both groups
		Very good	102 (36.7)	
		Good	98 (35.2)	
		Fair	48 (17.3)	
		Poor	8 (2.9)	
16	Overall mental / emotional health	Excellent	64 (22.8)	Question to both groups
		Very good	110 (39.2)	
		Good	72 (25.7)	
		Fair	31 (11.0)	
		Poor	3 (1.1)	

**Table 3:** Survey results, patient demographic data.

Item	Assessment question	Response parameter	Response	Explanation
17	Age (eight categories, minimum 18)	Mean category	55-64	Question to both groups
		Range	18 to > 85	
18	Gender	Male (n=106)	38.55	Question to both groups
		Female (n=167	60.73	
19	Education (six categories)	Mean category	Some college	Question to both groups

**Table 4:** Evaluation of hypotheses.

Hypothesis	Result	Interpretation
H1	z=15.24 *P*<.001 (Fisher’s exact test)	Strong support for H1, provider’s use of tablet/ software bundle to communicate information was perceived as helpful.
H2	z=13.21, *P*<.001 (Fisher’s exact test)	Strong support for H2, provider’s use of tablet/ software bundle made it easier to talk with provider.
H3	*t*=0.33, *P*=.74	H3 not supported, no age-related effect on perceived helpfulness of using tablet device to communicate was identified between low-age and high-age groups (divided at median age group).

### Results of the Study

These results provide strong and highly significant support for H1 and H2. We conclude that in the context of this study, the provider’s use of the tablet/software bundle to communicate information was helpful to patients. We also conclude that patients found the provider’s use of the tablet/software bundle made it easier for the provider and patient to communicate about medical information related to the patient’s treatment.

H3 was not supported. We conclude that acceptance and perceived helpfulness of the providers’ use of the tablet/software bundle was not affected by the age of the patient.

No differences between the groups were expected and none were found on items 1 through 9, as they strictly pertained to the provider, independent of the intervention. These questions were requested by the clinic in which the study was conducted, and they provide useful context to understand the study setting.

These results were independent of patients’ self-perceived physical or mental health, education, or gender, none of which were related to key outcomes.

### Qualitative Findings, Analysis of Physician Comments

Resident physicians who used the system in direct interaction with patients were asked to complete an online survey where they offered comments on the application of the tablet/instructional video combination, including typical uses, benefits, challenges, effect on patient satisfaction, and additional features that could improve the system, six months after patient data were collected. All five residents in the experimental group completed the survey.

eHealth applications have become more commonplace and are often developed based upon organizational or business goals. As mentioned earlier, there are a number of information systems that are used to improve communication with patients. The use of mobile discharge instruction videos is one strategy to improve communication with patients.

Residents stated that they used the tablets with instructional videos and 3D images in the outpatient adult clinic, and in the hospital during discharge or admission. To further elaborate, the following responses were received from the residents,

I had the patient watch the video while I was staffing with my preceptor. It helped that gap of time waiting. I also used it a few times in the hospital to help patients and families understand (diagnosis) better.

Resident

I can show (patient) images of anatomy of specific organ system when discussing and explaining to them their disease process.

Resident

Some of the residents stated that although they did use the tablet in the adult clinic, they believe that the tablet with instructional videos and 3D images are more useful in the emergency room and in-patient care facilities. Specifically, residents stated,

In general, I believe the videos are more beneficial in the ER, or even inpatient setting. I did not find them to be as useful in the outpatient setting where people are dealing with chronic illnesses.

Resident

More for in patient education. Not as appropriate for out patient. Used for diagrams of anatomy to explain illnesses.

Resident

...I do NOT believe the videos are appropriate for the clinic. I think they could be much better used on the in-patient side...

Resident

Used more frequently in-patient.

Resident

Video-based programs are among the most successful strategies to improve communication with patients [38,40], where it has shown a consistent increase in short-term knowledge, and have outperformed plain written materials, lectures, and individual counseling [41]. Residents reported numerous benefits to using the tablet with instructional videos and 3D images, including improved patient understanding, communication of diagnosis and treatment, and patient compliance. Specifically, the residents reported,

Some better understanding for my patients.

Resident

Very precise and clear.

Resident

...better patient visualization...

Resident

[I am] able to explain disease processes better by showing pt (patients) images of what I'm trying to explain.

Resident

I think the videos would be PERFECT for the setting of discharge from the hospital.

Resident

I think by showing pts (patients) what is going wrong in their body, rather than just telling them what is wrong, will make them more likely be compliance with medical care to improve their condition.

Resident

Residents further commented on how the tablet with instructional videos and 3D images impacted their interactions with patients. They stated,

More understanding about what I was trying to explain to them.

Resident

I didn't feel it impacted my interaction but I think they (patient) liked the videos.

Resident

It is a good tool to help augment (patient’s) understanding of their medical condition.

Resident

There are 100 instructional videos that the residents were able to use when treating patients, and although they are going to choose videos that are appropriate for the diagnosis they are treating, they reported that the table with instructional videos and 3D images are most beneficial for specific conditions. Specifically, the residents reported that they could pull up videos and/or images for,

Cardiac problems.

Resident

Diabetes.

Resident

CHF, smoking cessation, PE/DVT, anticoagulation (management), diabetes.

Resident

In patient, especially diverticulosis, gall bladder issues/surgery.

Resident

Back pain, I can pull up images of spinal column to explain different disease processes such as disc herniation or spinal stenosis.

Resident

The residents believe that patient satisfaction was improved with the use of the tablet with instructional videos and 3D images. When videos specific to the patient’s situation were available, “patients seemed to like the videos”.

Although the residents reported numerous benefits to using the tablet with instructional videos and 3D images, they also reported a few challenges, including allocating time needed to launch the system and locate appropriate videos. Specifically, the residents stated,

...because the clinic is such a fast paced environment, I do NOT believe the videos are appropriate for the clinic. I think they could be much better used on the in-patient side. It's to time consuming in clinic.

Resident

...mainly taking time to turn it on and searching for and bringing up the images or videos that I'd like to show.

Resident

While most of the residents reported that no additional features to the tablet with health care instructional videos and 3D images are needed, two of the residents suggested that, “More pictures (that are) easier to find”, and “More clinic-oriented videos (to help patients understand clinic-administered treatments) would held to increase patient understanding and satisfaction”.

In summary, physician responses reported comments on uses, benefits, challenges, satisfaction, and additional features, as follows,

Typical uses of the tablet/instructional video combination included: (1) use In the hospital during admission; (2) use in the hospital during discharge; (3) use in outpatient clinic; (4) used during hospital stay to help patients and families understand diagnoses, diagrams and illustrations of anatomy helped the physician explain illnesses; and (5) tablet/instructional video combination was found to be helpful to patients during time gap presented by waiting for the next test, exam, or procedure.

Benefits to use of tablet/instructional video combination included: (1) improved understanding by patients; (2) precision, clarity, and improved patient visualization; (3) ability to explain disease processes using images; (4) possible improved patient compliance, showing patients illustrations may make them more compliant with medical instructions; and (5) assistance in explaining illnesses and treatments in specific disease processes, including diabetes, heart failure, pulmonary embolism, deep vein thrombosis, anticoagulation management, diverticulosis, gall bladder disease and surgery, back pain, and smoking cessation.

Challenges to use of tablet/instructional video combination included: (1) time limitations in terms of allocating time from treatment tasks; and (2) time required to simply launch the system and locate appropriate topics.

Patient satisfaction was improved with use of the tablet/instructional video combination in at least two ways: (1) the combination system was seen as helpful by individual patients; and (2) when videos specific to the patient’s situation were available, patients “seemed to like the videos”.

Additional features to the tablet (with health care instructional videos and 3D images) that would help patient understanding included: (1) additional pictures and diagrams for patients; (2) more clinic-oriented videos (to help patients understand clinic-administered treatments); (3) additional medical topics should be added, explained by additional instructional videos; and (4) videos would be most useful in an emergency room environment or a hospital inpatient setting, as opposed to outpatient encounters that address chronic conditions.

## Discussion

### The Promise of Computer-Assisted Guidance for Physicians and Patients

The glowing promise of computer-assisted guidance, instruction, and explanation of medical conditions and treatment methods for patients has been recognized for decades. Since the introduction of handheld computer devices with easily accessible visual and audio interfaces, the ability of information and communication technologies to help physicians and other health care providers to communicate detailed technical and instructional information to patients has become ever more clear. Yet, the reality of limited testing of such technology tools in clinical settings has not yet supplied a satisfactory body of evidence that would justify widespread agreement on the technology’s potential as a successful educational and motivational approach for patients. Many important, open questions remain.

First, what is the pattern of initial patient reaction to use of such an instructional system? Technologies that employ computer interfaces and video recorded information have historically been a source of frustration and even annoyance to those who are not comfortable with use of technologies. Does exposure or familiarity help those who have had limited experience with computer-assisted instructional systems in the past? Initial reactions are not simply a function of age, as this study has shown. Responsiveness to technology-based guidance can also be a result of education, lifestyle, and even intelligence and personality factors.

Second, is the system easy to understand? How do we best understand patient comprehension of instructions in the context of use of computer-assisted guidance systems?

Third, how does such an independent, computer-assisted instructional system perform compared to direct, face-to-face interaction with a health care advisor? Is it comparable to the instructional method of sitting with a nurse who explains medications and bandage changing schedules? Is it superior to direct interaction in any way? Is it more efficient for health care providers to communicate essential information? Does its use improve patient safety? Can it substitute for or augment bedside interaction with a health care professional?

Communication skills and emotional awareness of health care providers are viewed as key aspects of professional competence [8]. Unfortunately, increasing pressures placed on medical providers to process patients in a minimal amount of time works against efforts to encourage improved communication between the provider and the patient [7]. Some providers may regard sensitivity and clarification in such communication as a luxury.

This study added to the body of evidence that computer-assisted instructional systems for patients can provide solid support for the overall objective of making sure patients understand instructions from their health care providers. As in a few recent studies, this research evaluated perceptions about the use of mobile devices in patient-provider communications. Several recent studies have focused on multimedia content provided through a computing device for patient educational purposes. However, few studies have combined both mobile devices and multimedia patient education in the same study. Further, unlike other studies on this topic, the research setting was a live field test, providing a real world, medical outpatient treatment environment to study patient perceptions. In addition, unlike other studies, this research included patient perceptions (quantitative) combined with physician perceptions (qualitative). The key findings of the study are: (1) Patients found the computer-based instructional system to be helpful. Although we did not test for openness and liking of the technology, it is clear that the measured helpfulness is clearly associated with an overall openness to the technology and the lack of intimidation produced in patients by the computer-based system. And (2) patients found that use of the computer-based instructional system made it easier to communicate with their health care providers. Clinical discharge environments can be stressful for all concerned, and time pressures are ever present for health care professionals. Any tool that can help them communicate with patients is useful. The fact that patients found that it made communication easier adds to the impression that they liked using the system, chronological age of patients, which ranged from 18 to over 85, did not affect any aspect of their perception of the computer-based instructional system. Thus, in this context, an age-based digital divide was not found. This is very encouraging, given that a large segment of the older population of the United States still has little experience with computers.

This research opens the door for continued exploration of the question of how computer-based instructional systems can be best designed and deployed to support the efforts of health care professionals on whom increasing time demands are made.

### Limitations

This study has several limitations. First, although the study sample size was reasonably large (N=284), the sample itself is a convenience sample of patients who were discharged over a limited period of time. The patient sample demographics and other characteristics may not reflect the attitude of patients in other parts of the country, or those outside the United States. A second limitation is that the training provided for physicians in use of the tablet-based instructional system was adequate, but was limited by the well-known time limitations and work demands placed on medical residents. More extensive training may lead to even better outcomes. Finally, while the comments from patients showed a positive and favorable response in most areas, our use of the validated CAHPS survey instrument gives us confidence in the results.

### Future Research Paths

This study opens the door to many possible pathways for interesting research. For example, the usability and readability of a small tablet screen may be enhanced by alternative form factors, including those of the larger screen devices that are currently coming available. In addition, greater personalization of the system instructions, such as adding the patient’s name, the health care providers’ names, and some details of the patient’s individualized medical treatment can be included. Another important area is message reinforcement; patients were not able to take the tablet device home with them, and the ability to do so may reinforce the understanding and retention of the treatment instructions and enhance the effectiveness of the system. Finally, special versions of the software can be developed for defined target groups, including underserved populations, non-English speakers, and groups with specific cultural requirements.

## Multimedia Appendix 1

Patient survey instrument.

## Multimedia Appendix 2

Medical resident survey instrument.

## Multimedia Appendix 3

List of instructional video topics available to physicians.

## Abbreviations

CAHPS: Consumer Assessment of Health Care Providers
EHR: electronic health records
IT: information technology
3D: three-dimensional
