# Supplementary material for: Patient-Provider Communications in Outpatient Clinic Settings: A Clinic-Based Evaluation of Mobile Device and Multimedia Mediated Communications for Patient Education
Source: JMIR Mhealth Uhealth. 2015 Jan 12;3(1):e2. doi: 10.2196/mhealth.3732 (PMC4319142; doi:10.2196/mhealth.3732)
Supplement: Supplementary file 1 [file mhealth_v3i1e2_app1.pdf]

Patient Understanding and Satisfaction Survey

This survey asks about you and the health care you received today. Please take the time to complete this survey as honestly as possible. Your answers are very important to us. NO information that identifies you will be disclosed.

What To Do When You're Done
Once you complete the survey, place it in the locked drop box located behind the nurses' station. If you want to know more about this study, please call xxx xxxxxx at xxx-xxx-xxxx.

Survey Instructions

Answer each question by marking the box to the left of your answer. You are sometimes told to skip over some questions in this survey. When this happens you will see an arrow with a note that tells you what question to answer next, like this:

Yes → **If Yes, go to #1 on page 1**
No

Your Provider

Our records show that you got care from the provider named below.

<Name of provider label goes here>

1. Is that right?

Yes
No

The questions in this survey will refer to the provider named in Question 1 as "this provider." Please think of that person as you answer the survey. Please answer only for your own health care.

Your Care from this Provider During Your Most Recent Visit

2. Wait time includes time spent in the waiting room and exam room. During your most recent visit, did you see this provider **within 15 minutes** of your appointment time?

Yes

No

Communication with Provider

3. During your most recent visit, did this provider explain things in a way that was easy to understand?

Yes, definitely

Yes, somewhat

No

4. During your most recent visit, did this provider listen carefully to you?

   Yes, definitely

   Yes, somewhat

   No

5. During your most recent visit, did you talk with this provider about any health problems or concerns?

   Yes

   No >> **If no, go to #7**

6. During your most recent visit, did this provider give you easy to understand instructions about taking care of these health problems or concerns?

   Yes, definitely

   Yes, somewhat

   No

7. During your most recent visit, did this provider seem to know the important information about your medical history?

   Yes, definitely

   Yes, somewhat

   No

8. During your most recent visit, did this provider show respect for what you had to say?

   Yes, definitely

   Yes, somewhat

   No

9. During your most recent visit, did this provider spend enough time with you?

   Yes, definitely

   Yes, somewhat

   No

10. Did this provider ever use a computer or handheld device to show you information?

    Yes

    No >> **If no, go to # 13**

11. Was this provider's use of a computer or handheld device helpful to you?

    Yes, definitely

    Yes, somewhat

    No

12. Did this provider's use of a computer or handheld device make it harder or easier for you to talk with him or her?

    Harder

    Not harder or easier

    Easier

Overall Rating - Provider

13. Would you recommend this provider's office to your family and friends?

- Yes, definitely
- Yes, somewhat
- No

14. Using any number from 0 to 10, where 0 is the worst provider possible and 10 is the best provider possible, what number would you use to rate this provider?

- 0 Worst doctor possible
- 1
- 2
- 3
- 4
- 5
- 6
- 7
- 8
- 9
- 10 Best doctor possible

About You

15. In general how would you rate your overall health?

- Excellent
- Very good
- Good
- Fair
- Poor

16. In general, how would you rate your overall **mental or emotional** health?

- Excellent
- Very good
- Good
- Fair
- Poor

17. What is your age?

- 18-24
- 25-34
- 35-44
- 45-54
- 55-64
- 65-74
- 75-84
- 85 or older

18. Are you male or female?

- Male
- Female

19. What is the highest grade or level of school that you have completed?

- 8th grade or less
- Some high school, but did not graduate
- High school graduate or GED
- Some college or 2-year degree
- 4-year college graduate
- More than 4-year college degree

No, not Hispanic or Latino

20. Are you of Hispanic or Latino origin or descent?

Yes, Hispanic or Latino

21.

22. What is your race: Please mark one or more.

White

Black or African-American

Asian

Native Hawaiian or other Pacific Islander

American Indian or Alaska Native

23. Did someone help you complete this survey?

Yes

No >> **Thank you. Please return the completed survey**

24. How did that person help you? (Please mark one or more.)

Read the questions to me

Wrote down the answers I gave

Answered the questions for me

Translated the questions into my language

Helped in some other way

Thank you for your participation!

Please place the completed survey in the locked drop box located behind the nurses' station. If you want to know more about this study, please call xxx xxxxxx at xxx-xxx-xxxx.
