# Supplementary material for: Patient-Provider Communications in Outpatient Clinic Settings: A Clinic-Based Evaluation of Mobile Device and Multimedia Mediated Communications for Patient Education
Source: JMIR Mhealth Uhealth. 2015 Jan 12;3(1):e2. doi: 10.2196/mhealth.3732 (PMC4319142; doi:10.2196/mhealth.3732)
Supplement: Supplementary file 2 [file mhealth_v3i1e2_app2.pdf]

Questions for the Resident Physicians

1. I believe the healthcare instructional videos and 3D images helped my patients understand their diagnosis and/or discharge instructions?

Level of Agreement
1 – Strongly disagree
2 – Disagree
3 – Somewhat disagree
4 – Neither agree or disagree
5 – Somewhat agree
6 – Agree
7 – Strongly agree

2. I believe that using the tablet computer with healthcare instructional videos and 3D images helped my patients understand their diagnosis and/or discharge instructions?

Level of Agreement
1 – Strongly disagree
2 – Disagree
3 – Somewhat disagree
4 – Neither agree or disagree
5 – Somewhat agree
6 – Agree
7 – Strongly agree

3. Is there anything you would change about the tablet with healthcare instructional videos and 3D images that would help to improve patient understanding? Please explain?

4. Is there anything you would change about the tablet with healthcare instructional videos and 3D images that would help to increase patient satisfaction? Please explain.

5. Please describe how you typically used the tablet with healthcare instructional videos and 3D images. Please explain.

6. What challenges did you experience when using the tablet with healthcare instructional videos and 3D images? Please explain.

7. What benefits did you experience by using the tablet with healthcare instructional videos and 3D images? Please explain.

8. What benefits do you believe the clinic experienced by using the tablet with healthcare instructional videos and 3D images? Please explain.

9. For which health conditions do you believe the tablet with healthcare instructional videos and 3D images were most beneficial? Please explain.

10. How did using the tablet with healthcare instructional videos and 3D images impact (change, alter, augment) interactions with your patients? Please explain.

11. To what extent do you believe using the tablet with healthcare instructional videos and 3D images could increase patient compliance with discharge instructions? Please explain.

12. What additional features to the tablet (with healthcare instructional videos and 3D images) do you believe would help to increase patient understanding? Satisfaction? Please explain.
