# Supplementary material for: Patient-Provider Communications in Outpatient Clinic Settings: A Clinic-Based Evaluation of Mobile Device and Multimedia Mediated Communications for Patient Education
Source: JMIR Mhealth Uhealth. 2015 Jan 12;3(1):e2. doi: 10.2196/mhealth.3732 (PMC4319142; doi:10.2196/mhealth.3732)
Supplement: Supplementary file 3 [file mhealth_v3i1e2_app3.pdf]

Topic	ER	Inpatient
ER Discharge	X	
1 Abdominal Pain	X	X
2 Ankle Sprain	X	
3 Back Strain	X	
4 Otitis Media	X	
5 UTI	X	X
6 Head Injury	X	X
7 Headache	X	
8 Seizure	X	X
9 Pneumonia	X	X
10 Pharyngitis	X	
11 Tooth Infection	X	
12 Bronchitis	X	
13 Cellulitis	X	X
14 Cervical Strain	X	X
15 Croup	X	X
16 Diarrhea	X	X
17 Febrile Seizure	X	
18 Lower Extremity Fracture	X	X
19 Upper Extremity Fracture	X	X
20 Upper Extremity Sprain	X	
21 Lower Extremity Sprain	X	
22 Abscess	X	X
23 Nose Fracture	X	X
24 Contusion	X	
25 Dislocation	X	X
26 Nose Bleed	X	X
27 Sinus Disease	X	
28 Dysfunctional Uterine Bleeding	X	X
29 Pelvic Infections	X	X
30 Pelvic Pain Unknown Cause	X	
31 Threatened Miscarriage	X	X
32 Asthma	X	X
33 Biliary Colic	X	X
34 Chest Pain Unknown Cause	X	X
35 Conjunctivitis	X	X
36 COPD/Emphysema	X	X
37 Corneal Injury	X	
38 Diverticulitis	X	X
39 Dysrhythmia	X	X
40 Renal Colic	X	X
41 Wounds	X	X
42 Viral Illness	X	
43 Vomiting	X	
44 Ulcer Disease	X	
45 Otitis Externa	X	
46 Completed Miscarriage	X	X
47 Constipation	X	X
48 Reflux Esophagitis	X	X
49 Allergic Reaction	X	X
50 Hypertension	X	X

51 Fever	X	X
52 Syncope	X	X
53 Vertigo	X	X
54 Bell's Palsy	X	
55 Hypoglycemia	X	X
56 Hyperglycemia	X	X
57 Substance Abuse	X	X
58 Pain	X	X
59 Dermatitis	X	X
60 Pyelonephritis	X	X
61 Tendonitis	X	
62 Withdrawal	X	X
Procedures		
63 Procedural Sedation	X	X
64 Lumbar Puncture	X	X
65 Incision and Drainage	X	X
66 Paracentesis	X	X
67 Stroke—tPA	X	X
68 Cardioversion		X
69 PTCA	X	X
70 Colonoscopy	X	X
71 Dobutamine Echo		
72 Stress Echo		
73 Angiography		
Diagnostics		
74 CT Scan	X	X
75 MRI	X	X
76 Ultra Sound	X	X
77 Nuclear Medicine		X
Medications		
78 Narcotics	X	X
79 Respiratory Inhalers	X	X
80 Antibiotics	X	X
81 Benzodiazepines	X	X
82 Antipyretics	X	X
83 Antihypertensives	X	X
84 Lipid Medications	X	X
85 Anticoagulants		X
86 Antiplatelets	X	X
Other		
87 Advance Directives	X	X
88 Smoking Cessation		X
89 Safety	X	X
90 Respiratory Failure/Ventilator		
Inpatient Discharge		
91 TIA		X
92 Heart Attack		X
93 CHF		X
94 PE		X
95 DVT		X
96 Angina (for ACS)		X
97 Sepsis		X
98 CVA		X
99 Post op Wound Care		X

100. Home Care:		
Emergency Care Plan		
